# Complementary role of cardiac CT in the assessment of aortic valve replacement dysfunction

**DOI:** 10.1136/openhrt-2016-000494

**Published:** 2016-11-02

**Authors:** Alastair J Moss, Marc R Dweck, John G Dreisbach, Michelle C Williams, Sze Mun Mak, Timothy Cartlidge, Edward D Nicol, Gareth J Morgan-Hughes

**Affiliations:** 1 Centre for Cardiovascular Science, University of Edinburgh, Edinburgh, UK; 2 Department of Radiology, Glasgow Royal Infirmary, Glasgow, UK; 3 Department of Radiology, Imperial College Healthcare NHS Trust, London, UK; 4 Department of Cardiology, Royal Brompton Hospital and Harefield NHS Trust, London, UK; 5 Department of Cardiology, Derriford Hospital, Plymouth, UK

## Abstract

Aortic valve replacement is the second most common cardiothoracic procedure in the UK. With an ageing population, there are an increasing number of patients with prosthetic valves that require follow-up. Imaging of prosthetic valves is challenging with conventional echocardiographic techniques making early detection of valve dysfunction or complications difficult. CT has recently emerged as a complementary approach offering excellent spatial resolution and the ability to identify a range of aortic valve replacement complications including structural valve dysfunction, thrombus development, pannus formation and prosthetic valve infective endocarditis. This review discusses each and how CT might be incorporated into a multimodal cardiovascular imaging pathway for the assessment of aortic valve replacements and in guiding clinical management.

## Introduction

The utility of cardiac CT for the assessment of possible aortic valve replacement dysfunction has risen rapidly over the past 10 years following a similar, albeit delayed, trajectory to CT coronary imaging. It can be used to assess mechanical and bioprosthetic valves inserted surgically as well valves inserted using transcutaneous aortic valve implantation (TAVI). Clinicians familiar with both cardiac CT and valvular heart disease have identified a number of specific situations where CT can help in the assessment of possible aortic valve replacement dysfunction by providing complementary diagnostic information to transthoracic echocardiography, transoesophageal echocardiography and cardiac MR. These include the identification of pannus formation, thrombus, premature bioprosthetic leaflet degeneration, assessment of bileaflet mechanical valve leaflet motion and aortic root abscess formation. While the role of cardiac CT is relatively new in this setting, its use is steadily expanding across the world, with many experienced centres now using it routinely. This article evaluates the evidence in support of cardiac CT imaging for the detection of possible aortic valve replacement dysfunction and aims to prompt clinicians to consider it in specific clinical scenarios.

## Bioprosthetic aortic valve replacement dysfunction

Selecting the appropriate prosthetic heart valve has traditionally been a difficult decision for many patients undergoing surgical aortic valve replacement. However, recent advances in bioprosthetic valve design, coupled with an ageing population, have witnessed increasing use of these valves in preference to metallic valves. With the recent addition of TAVI, the number of patients with functioning bioprostheses is only set to expand further. Surveillance of bioprosthesis function, looking for evidence of valve degeneration, forms an integral part of the long-term management of patients with these valves and a substantial healthcare burden.^1^ This continues to remain relevant in the modern era where newer generation bioprosthetic valves offer improved longevity but still have a limited life span of 10–15 years. Despite technological advances, bioprostheses exhibit more frequent structural valve dysfunction than mechanical valves (2.17%/patient-year vs 0%/patient-year, p=0.0001), resulting in higher rates of repeat operation (2.32%/patient-year vs 0.62%/patient-year, p=0.0003).^2^ The high spatial and temporal resolution of cardiac CT makes it well suited to visualise many of the complications that can arise following bioprosthetic valve implantation, namely, structural valve dysfunction, thrombus development, pannus formation and prosthetic valve infective endocarditis.^3^ We will here examine the role of CT in assessing each of these problems.

### Structural valve dysfunction

Structural valve dysfunction can have catastrophic consequences, yet its underlying pathophysiology remains incompletely understood. The term structural valve dysfunction encompasses intrinsic functional changes to the valve leaflets including retraction or tearing, progressive stenosis and disruption of the annular housing or sewing ring. The principal pathological driver behind this degeneration and eventual failure appears to be leaflet calcification. This most commonly results in valvular regurgitation due to tearing of the leaflets but may also cause increasing valve stiffness, restenosis and peripheral embolism.^4^ At the subcellular level, scanning electron microscopy has revealed that microcrystalline hydroxyapatite and amorphous calcium phosphate aggregate into plate-like structures that are incorporated into the collagen matrix.^5^ Calcification has been observed in over half of porcine prosthetic valves implanted 5 years previously, rising to over three-quarters of valves aged 8 years or more.^6^ ^7^ Interestingly valve calcification and degeneration appears accelerated in younger patients, perhaps due to increased mechanical stresses in these patients, as highlighted by the fatal complications following implantation of bovine pericardial bioprostheses in children and young adults.^8^ Indeed, such is the importance of calcification in structural valve dysfunction that efforts by valve manufacturers aimed at improving longevity have largely focused on anticalcific strategies including the application of topical anticalcific agents to the leaflet surfaces.

The mechanisms driving prosthetic valve calcification are incompletely understood with several different processes having been implicated.^9^ The combination of glutaraldehyde pretreatment and an immune rejection response to residual animal antigens in the bioprostheses leads to the accumulation of extracellular calcium.^10–13^ However, recent data have suggested a third potential mechanism with evidence supporting prosthetic valve calcification as an active disease process with many similarities to those observed in aortic stenosis and atherosclerosis.^13^ ^14^ CT is the technique of choice for imaging macroscopic deposits of calcium in the vasculature and so potentially allows leaflet calcification and degeneration to be identified at an earlier subclinical stage.

**Figure 1:**
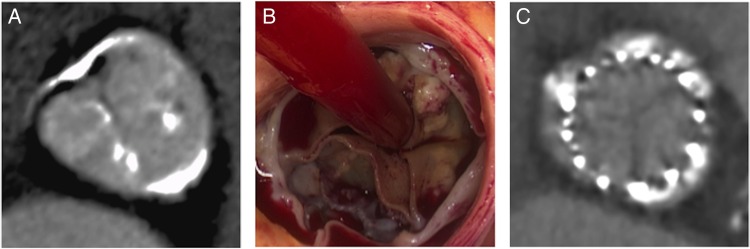
Structural valve degeneration. (A and B) Bioprosthetic aortic valves calcify along the cusp commissures mimicking the pattern found in native aortic valve disease. (C) Calcified leaflets are reflected onto the sinus walls during transcatheter valve implantation.

While large-scale clinical trials are currently lacking, the requirement for improved assessment of bioprosthetic valve calcification was recently illustrated in the case of severe bioprosthetic valve obstruction in a 13-year-old girl who died suddenly just 23 months after implantation of a bovine pericardial aortic bioprosthesis.^8^ The rapid deterioration in her valve function occurred despite recent echocardiography demonstrating only mildly stenotic gradients and restricted motion of a single leaflet. Ex vivo CT was performed on this and two other explanted bioprostheses demonstrating extensive calcification within the central part of the valve leaflets between the aortic and ventricular surfaces. Immunohistochemistry showed that these calcium deposits extended along collagen fibres within the leaflet thereby increasing leaflet thickness. In principle, calcium is best imaged on non-contrast CT scans, such as those used for CT calcium scoring of the coronary arteries and native aortic valves. However, using dedicated software, it is also possible to quantify leaflet calcification on contrast-enhanced cardiac CT, allowing improved localisation of calcific deposits to the bioprosthetic leaflets rather than surrounding structures.^15^ The challenge is deciding on a suitable threshold for identifying calcium on these scans, although a detection threshold of 850 Hounsfield units (HU) has recently been used to identify calcification in native valves that ultimately goes on to cause clinical complications following TAVI.^15^ Structural components within the prosthesis such as the stent frame and sewing ring may generate beam hardening artefact that may obscure or even be confused with bioprosthetic calcification. An appreciation of the different structural designs of the various bioprosthetic valves is therefore of use when trying to identify true structural valve dysfunction.^16^ While TAVI is currently reserved for high-risk surgical candidates with a reduced life expectancy, this procedure is increasingly used in high-risk younger adults where the long-term durability of these valves has not been evaluated. Reports of early TAVI valve degeneration have again implicated calcification as the key pathological driver, suggesting that CT may once again be off use in detecting early degeneration. This is supported by several recent observations. Deutsch and colleagues noted structural valve dysfunction with several areas of macrocalcification on CT in a 4-year-old CoreValve TAVI bioprosthesis implanted in to a 44-year-old.^17^ Similar findings have also been found in elderly patients early after valve implantation.^18^ ^19^ Importantly residual calcium from the original aortic valve around the perimeter of the TAVI bioprosthesis must be differentiated from new calcium formation within the leaflets of the new prosthetic valve (figure 1).

### Reduced leaflet mobility and hypoattenuation leaflet thickening

**Figure 2:**
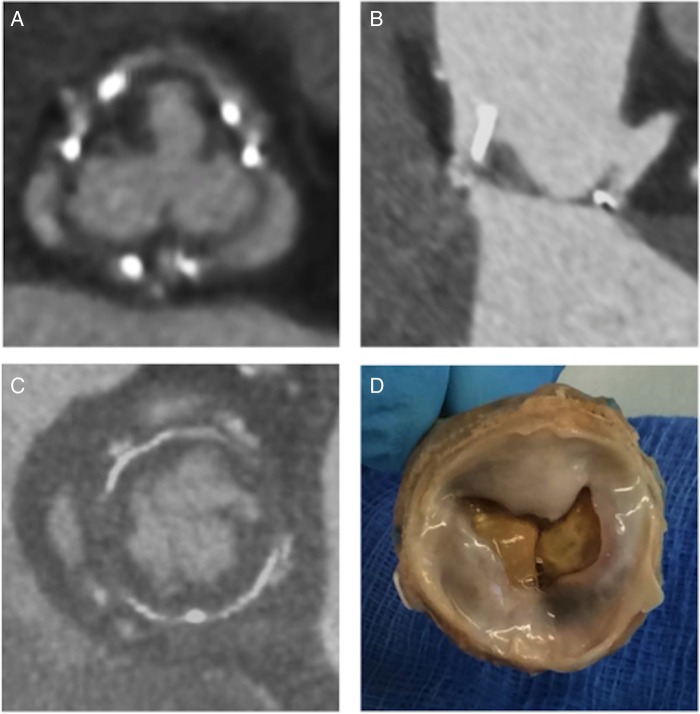
(A and B) Hypoattenuation leaflet thickening suggestive of thrombus formation on the supravalvular surface impinging leaflet motion. (C and D) Pannus formation underneath the prosthesis can also restrict leaflet motion necessitating surgical removal of the prosthesis.

One of the major advantages biological prosthetic heart valves have over mechanical alternatives is the lack of a requirement for long-term anticoagulant use. During bioprosthesis endothelialisation, in the first 3 months after implantation, thrombus formation can occur in 0.8–4.0% of cases.^20^ However, clinical sequelae of systemic thromboembolism and obstructive thrombosis are very rare phenomena occurring in 0.9% and 0.03% of cases, respectively.^21^ Recent studies performing contrast-enhanced cardiac CT in TAVI patients have identified a new phenomenon termed hypoattenuation leaflet thickening (HALT; figure 2). This was first described by Pache *et al*^22^ in an 86-year-old man with hypoattenuation of a single prosthetic cusp 7 days after implantation of a 29 mm SAPIEN XT valve. Subsequent resolution following coumadin anticoagulation suggested that this finding represented cusp thrombosis rather than pannus formation. Following this initial report, three more recent studies have further investigated the frequency and possible clinical significance of HALT,^23–25^ and each is discussed below.

Leetmaa *et al*^23^ reported the incidence of HALT in a consecutive cohort of 140 patients undergoing SAPIEN XT transcatheter aortic valve implantation. Using prospective-gated cardiac CT, five patients (3.6%) were found to have hypoattenuating masses on the aortic surface of the transcatheter valve at 3 months postimplantation. Combined data from the PORTICO IDE study as well as the RESOLVE and SAVORY registries have suggested a higher incidence of HALT (13–40% of patients) if scans are performed at earlier time points postimplantation (30 days to 3 months).^24^ In these pooled registries, reduced leaflet motion and hypoattenuated lesions were associated with an increased risk of systemic thromboembolism (3 of 17 patients with these abnormalities vs 1 of 115 patients without; p=0.007), with both features responding to therapeutic anticoagulation. Ex vivo modelling established that changes in mean pressure gradients are dependent on the size of valve and the number of cusps occluded. This may explain why these findings on cardiac CT are commonly observed in the absence of any increase in velocities or pressure gradient on echocardiography. Indeed at least two cusps must be involved in a 23 mm PORTICO valve for the effective orifice area to be sufficiently stenosed to meet current echocardiographic criteria for detection.^26^ Early CT assessment following SAPIEN 3 transcatheter valve implantation revealed hypoattenuated thickening in 10.3% of patients with a trend towards a higher incidence in patients administered single-antiplatelet therapy compared with dual-antiplatelet therapy.^25^ In 13 patients with hypoattenuated thickening who underwent repeat CT angiography after 3 months of treatment, all those on a combination of clopidogrel and phenprocoumon (WOEST regimen^27^) had complete resolution of CT abnormalities. Importantly, there were no thromboembolic or bleeding complications after 8 months of follow-up.

In light of the asymptomatic presentation of cusp thrombosis that occurs in spite of dual-antiplatelet therapy, important questions have been raised regarding the clinical relevance of hypoattenuated thickening and whether CT may more appropriately stratify antithrombotic therapy following transcatheter valve implantation. Importantly, the clinical significance of HALT has not been validated beyond these observational studies and the recent publication of low 30-day stroke rates following TAVI suggests that the presence of this finding does not always translate into significant thromboembolic events.^28^ Randomised control trials are warranted before recommending routine anticoagulation in a group of elderly patients that are at high risk of bleeding complications, although CT may find a role in pinpointing those patients with most to gain from this therapy.

## Mechanical aortic valve replacement dysfunction

Imaging of mechanical prosthetic valves has traditionally been performed using transthoracic echocardiography, transoesophageal echocardiography and fluoroscopy.^29^ ^30^ However, the anatomic evaluation of the annular housing, prosthetic valve leaflets and perivalvular structures by echocardiography has intrinsic limitations, most notably the acoustic shadowing artefacts produced by metallic components of the valve. Although by no means free of technical limitations, complementary roles for cardiac CT are emerging in the assessment of mechanical valves, particularly in acquired mechanical obstruction and endocarditis. While the metallic components of metallic valves also result in artefact on CT, diagnostic quality images can be obtained in the majority of mechanical valves.

### Acquired mechanical prosthetic valve obstruction—thrombosis and pannus formation

Acquired mechanical prosthetic valve obstruction (PVO) is an uncommon but serious and potentially fatal complication of valve replacement.^31^ A definitive diagnosis is of paramount importance due to the associated morbidity and mortality and the frequent need for appropriately timed intervention.^32^ The two main mechanisms of acquired mechanical PVO are thrombosis and pannus formation, both of which restrict normal leaflet motion.^31^ Differentiation of these two causes is of critical importance in guiding appropriate treatment (table 1). While both can be treated with urgent surgical revision, valve thrombosis is also potentially amenable to thrombolysis.^33^ Moreover accurate assessments of the degree of valve obstruction and the size of the thrombus will help to determine the urgency with which intervention is required alongside standard clinical assessments.^31^ CT is increasingly being used in this role to complement standard imaging and to help guide patient management, in particular when a clear aetiology has not been established.^30^

**Table 1:** Differentiating pannus from thrombus on cardiac CT

	Pannus	Thrombus
Timing of presentation	Usually 12 months after surgery	Occurs at any time
Location	Below the aortic prosthesis	Above or below the aortic prosthesis
Morphology on CT	Circular mass extending from sewing ring Contrast enhancement Calcification may be present	Irregularly shaped mass attached to leaflet or hingepoint No contrast enhancement
CT attenuation	>145 HU	<145 HU

HU, Hounsfield units.

Cardiac CT in suspected acquired mechanical PVO enables evaluation of leaflet opening and closing angles, dynamic leaflet motion and the composition of perivalvular masses valve helping to differentiate between valve thrombosis and pannus formation.^33^ A recent prospective trial compared the imaging results from cardiac CT with post-thrombolysis imaging and/or surgical findings in patients with acquired mechanical PVO.^33^ Of the 39 patients with a periprosthetic mass visible on cardiac CT, thrombus demonstrated a mean attenuation value of 87 (±59 HU) compared with 322 (±122 HU) for pannus. The investigators recommended a cut-off point of >145 HU for differentiating pannus from thrombus, providing a sensitivity of 88% and specificity of 96%. Additionally, in the patients who underwent thrombolysis, those with the lowest attenuation masses demonstrated the best response to thrombolysis with complete dissolution in all the <90 HU masses compared with just 42% in the masses with attenuation of between 90 and 145 HU.^33^

## Aortic valve replacement infective endocarditis

Prosthetic valve infective endocarditis carries a very high in-hospital mortality rate of 20–40%.^34^ Transthoracic echocardiography and transoesophageal echocardiography are the front-line investigations in the assessment of suspected prosthetic valve infection endocarditis;^29^ however, cardiac CT can prove useful particularly in cases where echocardiography is inconclusive.^32^ ^35^ Furthermore, emerging data suggest it can provide superior detection to echocardiography of several specific complications including perivalvular abscesses, pseudoaneurysms, valve dehiscence and extracardiac foci of infection.^35–38^ While echocardiography is the modality of choice for detecting vegetations and valve dehiscence, the complementary assessment of aortic root involvement provided by CT improves the diagnostic accuracy for planning surgical intervention (κ statistic 0.66–0.79 for CT or echocardiography alone vs κ statistic 0.88 for combined testing).^37^

**Figure 3:**
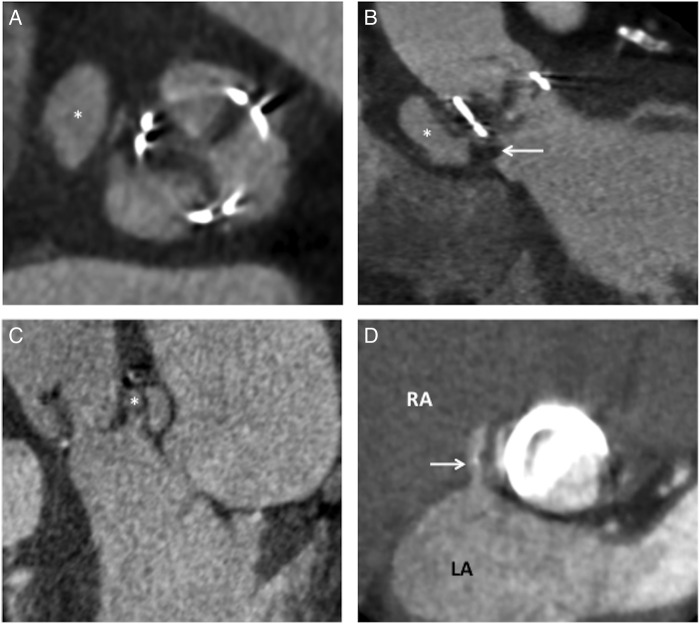
Prosthetic valve infective endocarditis. (A) Complications of prosthetic valve infective endocarditis include the development of perivalvular pseudoaneurysm formation (white asterix). (B) Saccular pseudoaneurysms (white asterix) occur in regions where vegetations (white arrow) erode through the annulus with a loss of sewing ring integrity. (C) Bacterial spread into the aortic root results in abscess formation (white asterix). (D) The anatomical location of erosive shunts (white arrow) and paravalvular leaks due to suture dehiscence can be readily identified using cardiac CT. LA, left atrium; RA, right atrium.

While the temporal resolution of echocardiography often means that highly mobile vegetations are better visualised on echocardiography, large prosthetic valve vegetations can be readily seen on CT as microlobulated, hypoattenuating lesions attached to the leaflets or sewing ring.^39^ If the infection spreads to involve the sewing ring, suture dehiscence can result in a paravalvular leak where there is a breach between the inflow and outflow tract. Loss of the sewing ring integrity leads to perivalvular pseudoaneurysm formation, which is evident as a focal, contrast-filled saccular or fusiform out-pouching arising from the annulus (figure 3). Extension of infection into the aortic root is more easily detected using CT compared with transoesophageal echocardiography and can further inform surgical management by detecting ancillary features such as pulmonary septic emboli, patency of the coronary arteries, mediastinal gas and collections.^38^ Importantly, the addition of CT imaging to standard assessments has been shown to change the clinical treatment strategy in a quarter of patients with suspected prosthetic valve infective endocarditis.^35^

## Technical considerations

In 2014, Ghersin *et al*^40^ published a suggested protocol for the CT evaluation of suspected prosthetic valve dysfunction. A retrospective contrast-enhanced cardiac CT is performed as the primary diagnostic scan for mechanical and bioprosthetic aortic valves. Scans acquired retrospectively can be reconstructed to provide images of the valve at multiple points across the cardiac cycle, allowing assessment of its open and closed positions, as well as the visualisation of dynamic motion through systole and diastole.^40^ The field of view can be limited to cover the level of the valve alone (reducing radiation exposure) or, depending on the clinical indications, expanded to cover other areas of interest, including the coronary arteries.

Multiple factors can affect image quality when assessing mechanical aortic valve prostheses using CT, in particular the presence of cardiac arrhythmia and older prosthetic valve types.^41^ Anecdotal evidence suggests that modern low profile bileaflet mechanical valves can be imaged with diagnostic certainty by cardiac CT with minimal beam hardening artefacts. Postprocessing techniques support the assessment of mechanical aortic valve leaflet opening and closing angles, limitation of leaflet motion and the presence or absence of periprosthetic pannus.^41^ The presence of a concurrent prosthetic mitral valve and variations in tube voltage ranging from 80 to 140 kV does not have a discernable affect on image quality.^41^ Minimising radiation dose while maintaining diagnostic quality images can be achieved with tube current modulation and the use of 100 kV tube potential in patients with a body mass index under 30 kg/m^2^.^40^

**Figure 4:**
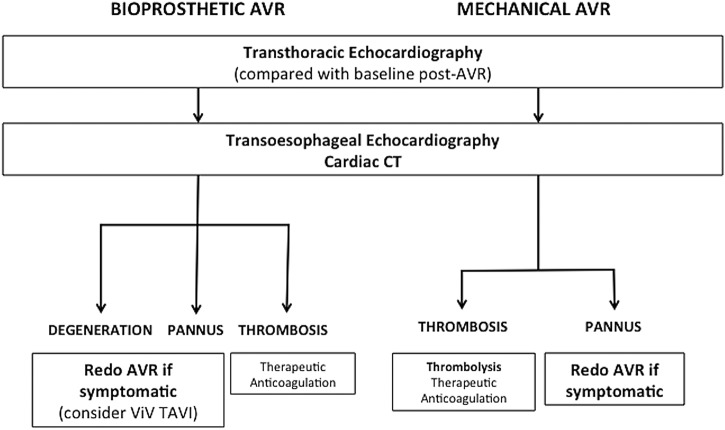
Suggested pathway for assessment of aortic valve replacement dysfunction. Transthoracic echocardiography with follow-on transoesophageal echocardiography and cardiac CT provides a detailed functional and anatomical assessment to guide further management. AVR, aortic valve replacement, ViV TAVI, valve-in-valve transcatheter aortic valve implantation.

**Figure 5:**
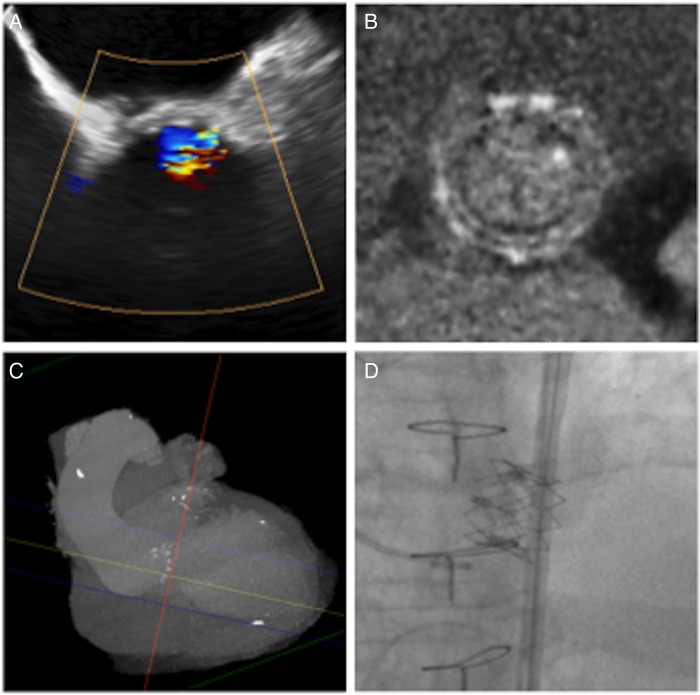
An example adjunctive CT imaging in the management of aortic valve replacement dysfunction. (A) Severe prosthesis regurgitation on transoesophageal echocardiography resulted from calcific structural valve dysfunction of the bioprosthetic leaflets in a 27 mm Aspire bioprosthesis demonstrated on CT (B). (C and D) Preprocedural planning facilitated implantation of a transcatheter aortic valve in valve.

Based on current practice in experienced valve centres, we suggest that CT should be performed as an anatomical assessment following transthoracic echocardiography in patients with suspected prosthetic aortic valve dysfunction (table 2, figures 4 and 5). The detailed structural assessment afforded by cardiac CT more accurately identifies the underlying pathology and facilitates the implementation of appropriate aortic valve intervention.

**Table 2:** The complementary role of CT in the assessment of aortic valve replacement dysfunction

	CT	Echocardiography
Prosthetic valve pathology		
Calcification	+++	+
Pannus	++	+
Thrombus	++	+
Vegetations	+	+++
Leaflet perforations/tears	−	+++
Valve dehiscence	+	+++
Paraprosthetic regurgitation	−	+++
Aortic root pathology		
Perivalvular abscesses	+++	++
Pseudoaneurysm	+++	+
Prosthetic valve function	+	+++
Coronary artery anatomy	+++	−
Presurgical planning	+++	+
Prevalve-in-valve planning	+++	+

## Conclusion

The diagnosis and management of aortic valve replacement dysfunction remains a significant clinical challenge. Cardiac CT provides complementary assessments of these valves allowing detection of structural dysfunction, leaflet calcification, thickening, thrombus and pannus formation. This can help to stratify downstream management and clinical decision-making as part of multimodality imaging approach.
